# Microbial Similarity between Students in a Common Dormitory Environment Reveals the Forensic Potential of Individual Microbial Signatures

**DOI:** 10.1128/mBio.01054-19

**Published:** 2019-07-30

**Authors:** Miles Richardson, Neil Gottel, Jack A. Gilbert, Simon Lax

**Affiliations:** a Department of Systems Biology, Columbia University, New York, New York, USA; b Integrated Program in Cellular, Molecular, and Biomedical Studies, Columbia University, New York, New York, USA; c Department of Pediatrics, University of California San Diego, La Jolla, California, USA; d Physics of Living Systems, Department of Physics, Massachusetts Institute of Technology, Cambridge, Massachusetts, USA; CEH-Oxford

**Keywords:** built environments, microbial ecology, microbial transmission

## Abstract

The microbiota of the built environment is an amalgamation of both human and environmental sources. While human sources have been examined within single-family households or in public environments, it is unclear what effect a large number of cohabitating people have on the microbial communities of their shared environment. We sampled the public and private spaces of a college dormitory, disentangling individual microbial signatures and their impact on the microbiota of common spaces. We compared multiple methods for marker gene sequence clustering and found that minimum entropy decomposition (MED) was best able to distinguish between the microbial signatures of different individuals and was able to uncover more discriminative taxa across all taxonomic groups. Further, weighted UniFrac- and random forest-based graph analyses uncovered two distinct spheres of hand- or shoe-associated samples. Using graph-based clustering, we identified spheres of interaction and found that connection between these clusters was enriched for hands, implicating them as a primary means of transmission. In contrast, shoe-associated samples were found to be freely interacting, with individual shoes more connected to each other than to the floors they interact with. Individual interactions were highly dynamic, with groups of samples originating from individuals clustering freely with samples from other individuals, while all floor and shoe samples consistently clustered together.

## Abstract (precis)

Humans leave behind a microbial trail, regardless of intention. This may allow for the identification of individuals based on the “microbial signatures” they shed in built environments. In a shared living environment, these trails intersect, and through interaction with common surfaces may become homogenized, potentially confounding our ability to link individuals to their associated microbiota. We sought to understand the factors that influence the mixing of individual signatures and how best to process sequencing data to best tease apart these signatures.

## INTRODUCTION

Numerous recent studies have uncovered the extent to which humans influence the microbial ecology of the spaces they occupy through microbial exchange between skin and the built environment. Most of these studies have focused on home-associated microbial communities (1–4), with home size, number of occupants, and building materials differentiated between sampling locations. Each of those confounding factors may have a significant impact on microbial community structure, and they are difficult to disentangle. Other studies have focused instead on the microbial ecology of public spaces, such as classrooms and hospital entrance halls (5–11). Although they have been able to demonstrate that most of the taxa colonizing those spaces are skin-associated taxa, they are unable to link individual human microbial signatures to their data.

Individuals create their own “microbial cloud” (12) by constantly shedding their own microbiota (13, 14). Individuals shed around 30 million bacterial cells per hour (13), and thus leave behind a “microbial fingerprint” which has been shown to be stable over time (15, 16), although body sites vary in their stability (17). Microbial flow in the built environment is a keen topic of interest, as human skin is the dominant contributor to the microbiome of built environments (18). Cohabitation of multiple individuals has been shown to influence the microbiota of common spaces and of the constituents themselves (3, 8, 11), and common areas may serve as mechanisms of microbial exchange between individuals (19, 20). Dormitory buildings, which have a standardized architectural design, common building materials and furnishings in the rooms, and even a common ventilation system, represent an intriguing model system in which to characterize the direct effects of an individual’s skin microbiota on their surroundings and to further elucidate the forensic potential of skin microbial signatures. In one sense, dorm rooms represent a number of replicates that can be used to uncover general patterns of human microbial exchange with the built environment. In a different sense, they are a “metacommunity” in which it is possible to record a network of interaction by logging visits between rooms and the use of common spaces. The divide between private rooms and common spaces such as hallways, lounges, and restrooms further enables us to tease apart individual microbial signatures in shared spaces.

Identifying microbial signatures relies on recovering individual-specific taxa, either through the use of universal markers such as the 16S gene (21), clade-specific markers (22, 23), or metagenomic information (24). It is unclear how methodological differences in sequence clustering impact the ability to link individuals to their surroundings through microbial similarity. To determine how to optimize the inference of individual microbial signatures, we employed three sequence processing methods to determine which was most discriminative in characterizing individuals. It has been observed that in many built environment studies, a large fraction of reads are attributed to a small number of operational taxonomic units (OTUs) (3, 11, 25). These OTUs come from a small selection of skin-associated taxonomic groups, including corynebacteria, staphylococci, pseudomonads, and streptococci (26, 27). As much of the differentiation between individuals occurs within a small number of taxonomic groups, it is unclear how to optimize sequence clustering for forensic inference, as OTU clustering may lump together similar sequences by design. OTU clustering is commonly used as a way to control for error introduced during sequence processing and sequencing, which can produce artifacts that obscure the true composition of a sample (28, 29). OTU clustering commonly occurs at the 97% similarity level, as this roughly corresponds to species identity (30). Among the most commonly used pipelines for OTU clustering is UPARSE (31), a method that constructs OTUs by prioritizing highly abundant unique sequences during clustering, as these highly abundant sequences are less likely to be sequencing errors.

At the same time, OTU clustering has limits, as the 97% threshold erases significant differences within closely related taxa and can overestimate similarity between taxa (32, 33). To overcome these limits, a number of methods have been introduced to determine exact sequences without clustering (34–37). DADA2 (36) is a reference-free sequence-based algorithm that separates sequence errors from biological variation based on an Poisson error model, which partitions reads into unique sequences by grouping together sequences with a high likelihood of sequencing error. In contrast, minimum entropy decomposition (MED) (35) is an unsupervised version of oligotyping (34), a method that iteratively partitions sequences based on Shannon entropy. A sequence alignment is generated, and nucleotide sites with high nucleotide variation are used to partition groups of sequences, which then proceeds iteratively within each partition until no sites contain sufficient variation to merit further decomposition. Oligotyping has been used to explore variation in host-associated bacteria (38, 39) and uncover bacterial ecotypes (40, 41). The increased ability to discriminate between closely related taxa, such as in *Blautia* found in sewage systems (42), allows for the identification of their hosts.

To explore the divide between public and private, we sampled 37 participants and their rooms from floors five through eight of the University of Chicago’s eight floor South Campus residence hall, with four time points over 3 months. Participants were drawn from one “house” in the dormitory, which serves a subset of the dormitory floor plan with shared common space and bathrooms. From participants, we swabbed both the skin of their dominant hand, and their personal effects, such as bed sheets and shoes. Additionally, common surfaces on each floor, including tables and bathrooms, were also sampled. Dorm rooms and common spaces had openable windows, along with forced air heating and cooling. Together, this collection of surfaces encompasses the divide between private and public space in the dormitory. Further, to determine the most effective 16S rRNA sequence processing method to enable individual identification, we employed UPARSE at 97% identity, DADA2, and MED on these collected data.

## RESULTS

### Clustering methodology impacts the success of forensic inference.

**FIG 1:**
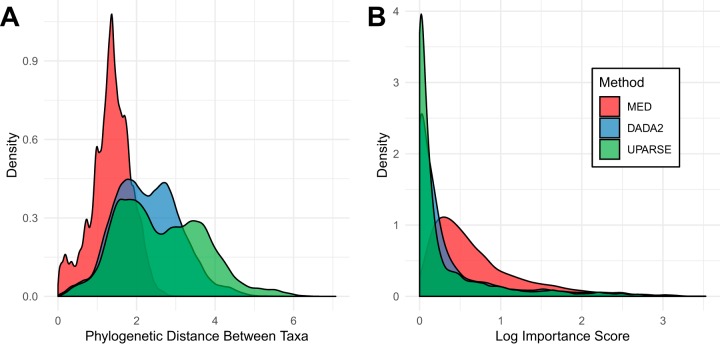
(A) Distribution of phylogenetic distance, based on the pairwise phylogenetic branch length between all taxa by each sequence processing method. MED recovers more highly related taxa than DADA2 or UPARSE. (B) Distribution of importance scores over all taxa, grouped by sequence processing method. The *y* axis is log transformed to aid visualization.

Each of the sequence processing methods produced a different picture of the microbial diversity of the dormitory. UPARSE recovered the largest number of distinct sequences (6,011) along with the greatest number of phyla (25 phyla). DADA2 recovered nearly the same phylum level diversity as UPARSE (23 versus 25) but fewer sequences (4,307). MED recovered fewer sequences (3,353) and fewer phyla (9 phyla) but recovered more members within each phylum (see Table S1 in the supplemental material). MED also had a significantly smaller phylogenetic distance between taxa (Wilcoxon rank sum test, *P* < 2.2e−16) than both DADA2 and UPARSE (Fig. 1A), indicating that MED recovered much more closely related sequences.

10.1128/mBio.01054-19.7 TABLE S1 OTU abundance and phylum-level diversity of each method. Download Table S1, DOCX file, 0.01 MB. Copyright © 2019 Richardson et al. 2019 Richardson et al. This content is distributed under the terms of the Creative Commons Attribution 4.0 International license.

**FIG 2:**
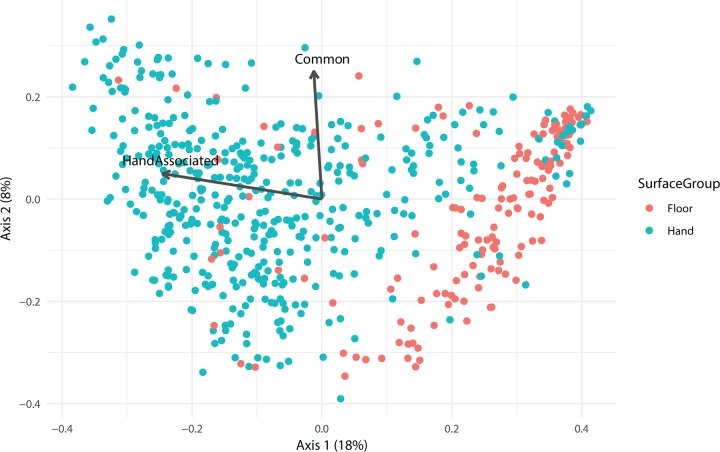
A principal component analysis (PCoA) plot based upon the Bray-Curtis distance. Statistically significant environmental vectors (*P* < 0.01) by *envfit* are plotted over the data. Common surface (*R*^2^ = 0.0654, *P < *10^−6^) and hand-associated (*R*^2^ = 0.38, *P < *10^−6^) vectors are shown.

Since we were most interested in classifying individuals, we compared each method using a random forest model trained on surfaces that closely associate with the hands of only one individual in order to test their forensic inference. There is a major divide between floor- and hand-associated samples (Fig. 2). Floor-associated samples, including shoes and floors, inhabit a different space compared to hand-associated samples, and this division significantly structures these communities (analysis of similarities [ANOSIM] on Bray-Curtis distance, *R* = 0.2821, *P* = 0.001). Thus, to predict which individual’s hands a surface had interacted with, bed sheets, desks, and door handles of the participant rooms are the most useful.

These models were implemented using a random forest model (43), which allows for the interrogation of similarity between samples. The model was then tested on hand samples from the same individuals, with the resulting accuracy summarized in Table 1. The standardized method of interpreting the success of classifiers is the error ratio, which quantifies how well the random forest model does at predicting the correct individual relative to the success expected by chance (44). An error ratio above two is commonly used as a significance threshold, and a higher ratio indicates better performance. All methods performed significantly better than random, but MED clearly outperformed UPARSE and DADA2 in our data set. Figure S1 in the supplemental material presents the confusion matrix generated by MED. Samples that fall on the diagonal are correctly classified by the random forest model. Most samples (79.57%) fall on the diagonal of the plot. However, for certain individuals, their hand samples are misclassified in every instance.

**TABLE 1:** Random forest model accuracy and error ratios

Method	Accuracy (CV-10) (%)	Error ratio
UPARSE	60.96	2.49
DADA2	71.06	3.36
MED	79.57	4.76

10.1128/mBio.01054-19.1 FIG S1 A confusion matrix generated by the results of a random forest model. It compares the actual identity of a sample with the one assigned by the random forest model. Accurate classification appears on the diagonal, and any deviation is an incorrectly predicted sample. Most samples fall on the diagonal, reflecting the 4.76 error ratio. Download FIG S1, PDF file, 0.01 MB. Copyright © 2019 Richardson et al. 2019 Richardson et al. This content is distributed under the terms of the Creative Commons Attribution 4.0 International license.

Interestingly, the largest source of classification error was the presence of roommates in the room. In fact, the classification error of an individual was linearly related to the number of roommates that individual had (*R*^2^ = 0.3143, *P < *0.0001), with classification error increasing by 18 percentage points for each additional roommate. The relationship is shown in Fig. S2. The random forest model attempts to use differences in taxon abundance between individuals to classify individuals. If two individuals interact and exchange bacteria, differences in abundance decrease, which in turn increases model error. Roommates had a significantly smaller weighted UniFrac distance between them than individuals residing in different rooms (Wilcoxon rank sum test, *W* = 409660000, *P* < 2.2 × 10^−16^).

10.1128/mBio.01054-19.2 FIG S2 Classification error plotted against the number of roommates. Download FIG S2, PDF file, 0.01 MB. Copyright © 2019 Richardson et al. 2019 Richardson et al. This content is distributed under the terms of the Creative Commons Attribution 4.0 International license.

### Classification of individuals is driven by specific taxa.

The random forest model is able to rank individual sequences or OTUs by their importance to successful classification. During the random forest generation process, only two thirds of variables are used to generate each forest. The accuracy of forests containing a given bacterial sequence can be compared to those without the sequence, and this is used to calculate the importance score. MED recovers significantly higher important scores than DADA2 or UPARSE and has a distinct distribution as seen in Fig. 1B. Furthermore, MED has a significantly higher average importance score (Wilcoxon rank sum test, false-discovery rate [FDR] *P* < 0.05) (Fig. S3) across all phyla that overlap between all three methods except for *Cyanobacteria*, *Fusobacteria*, and *Deinococcus*-*Thermus*.

10.1128/mBio.01054-19.3 FIG S3 Distribution of importance scores by phylum, with both Kruskal-Wallis significance between all pairs in the top of each panel, and Wilcoxon test results of means compared to MED importance scores on each bar. Download FIG S3, PDF file, 0.8 MB. Copyright © 2019 Richardson et al. 2019 Richardson et al. This content is distributed under the terms of the Creative Commons Attribution 4.0 International license.

It has been noted that there are taxa indicative of different sexes (45). To see whether there were enriched taxa between men and women from room samples, we looked for differentially enriched taxa using DESeq2. The most significantly enriched taxon was Lactobacillus iners, an inhabitant of the female reproductive tract. Certain corynebacteria were also noted to be more abundant in men, as seen in Fig. S4. Using these enriched taxa, we used the random forest model to predict whether a subject is a man or woman, with an error ratio of about 2.5, and accuracy of around 80% on the test set.

10.1128/mBio.01054-19.4 FIG S4 Differential abundant sequences between male and female individuals, with women at left and men at right. Download FIG S4, TIF file, 0.1 MB. Copyright © 2019 Richardson et al. 2019 Richardson et al. This content is distributed under the terms of the Creative Commons Attribution 4.0 International license.

### Metacommunity structure.

**FIG 3:**
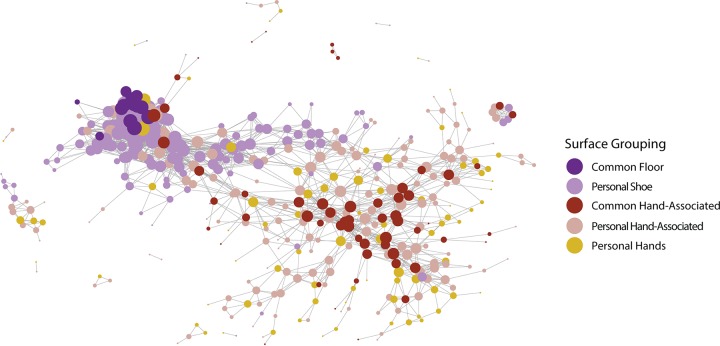
A weighted UniFrac graph of all samples, thresholded to be below 0.12 weighted UniFrac distance between individuals. They are sized based on their degree centrality, a measure of the number of connections they have to other samples. Samples are colored by sample type, with desks, bed sheets, and door handles grouped together as personal hand-associated samples. Common hand-associated surfaces act as a scaffold, connecting between themselves, along with connecting many distinct individuals.

In addition to classifying individuals, we sought to recapitulate the geographical structure of the dorm using graphical models. To do this, we constructed a threshold graph of the weighted UniFrac distance between samples, with edges preserved if they were less than a threshold of 0.12. As seen in Fig. 3, the dorm has two large subgraphs, along with a number of orphaned graphs. These two groups consist of floor-associated (shoes and floors) and hand-associated (hand, doorknob, bed, and desk) samples. The orphaned graphs are mostly samples from one individual. As expected, common surfaces in the hand-associated realm serve as an anchor for their subgraph, connecting a number of different people, while hallway floors serve the same role for individual shoes. In contrast, orphaned graphs appear to indicate the stability of an individual’s microbial signature over time and a lack of interaction with other samples.

While a graph can be constructed using a beta-diversity metric (in our case weighted UniFrac distance) as above, the distance metric may not be sensitive to the microbial community of an individual. Since there is information to be gained from aggregating samples into a larger individual signature, we also constructed a graph using random forest model proximity. The proximity values from the random forest model are akin to distance and take into account the same signature used to classify individuals. It is also much sparser, as the random forest model tries to minimize distances between samples from the same individual, while keeping samples between individuals distinct. The resulting graph can be seen in Fig. S5a and S5b, where samples are colored by individual and surface type, respectively. While these graphs show rough clustering by individual, and less clustering by surface, it is unclear at what level to delineate spheres of interaction. To compare how metadata related to both the weighted UniFrac and random forest graphs, we calculated the assortativity of various metadata.

Assortativity is a metric used to quantify how often nodes in a graph attach to other similar nodes, ranging from −1 to 1. Positive assortativity reflects high connectivity between similar nodes, while negative assortativity indicates connections between dissimilar nodes, with zero indicating no relationship. As seen in Table 2, all metadata factors had positive assortativity, with small positive assortativity values across the weighted UniFrac graph. The highest values belonged to the identity of a given surface and its personal or common nature, which implied that similar surfaces may share similar bacteria. Time point had low assortativity across graphs, indicating that the dorm has stable signatures over time. By contrast, the random forest graph had higher assortativity measures for floor, sex, and subject identity than the weighted UniFrac graph. The random forest is trained to distinguish individuals and their signatures, and the higher subject ID assortativity showed that it was better able to connect samples from unique individuals to each other. The increased assortativity of floor and sex may be related to this, as samples from one individual were also from the same floor and the same sex. At the same time, the assortativity of floor and sex were higher than subject ID, which may indicate that their association is more than just a result of grouping by subject. In addition to metadata, we wished to understand the spheres of interaction in the dormitory.

**TABLE 2:** Assortativity of metadata factors

Graph	Floor	Personal vs common	Sex	Surface	Time point	Subject ID
Weighted UniFrac	0.0496	0.2062	0.0583	0.1734	0.0705	0.0687
Random forest	0.3941	0.1446	0.3932	0.1985	0.0587	0.3245

10.1128/mBio.01054-19.5 FIG S5 Random forest model proximity graph colored by individual (A) or colored by surface type (B). Download FIG S5, PDF file, 0.2 MB. Copyright © 2019 Richardson et al. 2019 Richardson et al. This content is distributed under the terms of the Creative Commons Attribution 4.0 International license.

**FIG 4:**
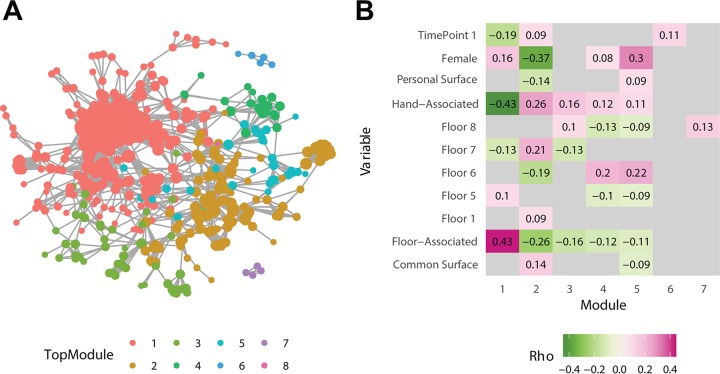
(A) A graph generated using random forest model proximity scores, trained to distinguish individuals. It is thresholded by proximity less than 0.076. It is colored by Top Module, the highest-level clustering produced by Infomap. Module 1 is mostly composed of shoe and floor samples, similarly to that shown in Fig. 3. (B) Significant Spearman correlations (*P* < 0.05) between each module and various metadata categories.

Graph-based clustering analysis methods are often used in describing interactions in social networks. Using the Infomap clustering algorithm (46), which uses flow within a network to generate groupings, we looked at how samples clustered into spheres of interaction. The relevant scale of interactions is not always clear, and the Infomap algorithm is also hierarchical (47), allowing for clustering of samples at many scales. This allows for samples to be first classified into large clusters, known as “top modules,” and then into smaller clusters within each top module, known as submodules, which are smaller groupings of fewer samples. Using this algorithm, we identified eight top modules (Fig. 4A), with module 1 encompassing almost all shoe and floor samples. We also wished to understand how these clusters related to the samples themselves and what factors associate with this structuring. As seen in Fig. 4B, a number of factors were significantly correlated with each cluster. Cluster 1, composed mostly of shoe samples, was associated with floor samples, and negatively associated with hand samples. In contrast, modules 2 through 4 all showed association with hand samples, while module 2 was more male and more related to common spaces than other modules. Further, we wished to examine which surfaces most commonly connect these spheres of interaction, and we found that hands were significantly enriched in connections between modules compared to the larger data set (binomial test, *P = *8.69 × 10^−8^). Other hand-associated surfaces showed enrichment, including common tables, doors, and bathroom doors (Fig. S6).

10.1128/mBio.01054-19.6 FIG S6 Bar graph comparing the proportions of samples connecting top modules compared to those in the data set at large. Hands show significant enrichment in connecting modules, indicating that they are the likely source of exchange between modules. Download FIG S6, PDF file, 0.01 MB. Copyright © 2019 Richardson et al. 2019 Richardson et al. This content is distributed under the terms of the Creative Commons Attribution 4.0 International license.

**FIG 5:**
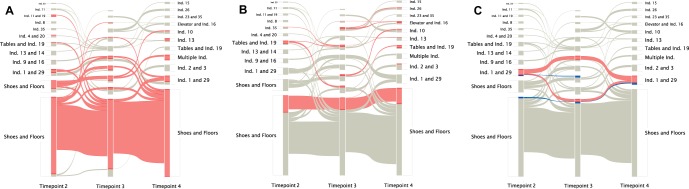
Alluvial diagrams depicting the clustering of samples over time. (A) All samples that were associated with the floor (hallway floors, bedroom floors, and shoes) were colored red. Ind., individual. (B) All common surfaces desks, bathroom doors, elevator buttons, and hallway floors. (C) Individual 1 (red) and individual 29 (blue) are indicated in color.

Of particular interest was how samples grouped over time, as samples that stably group together may indicate association. As we sampled the dorm over multiple discrete time points, we have a number of separate interaction graphs at each time point. This can be expressed as a multilayer graph (48, 49), where each time point is its own graph, representing interactions at a single point in time. These separate graphs are also connected by interactions which occurred between samplings, which we can estimate using the distances between these samples. To account for this structure, we employed a multilayer implementation of the Infomap algorithm to look at the stability of interactions over time (50). Here, we used samples from time points 2 to 4, as time point 1 consisted of only samples from participant hands and was not directly comparable to the other three time points. This is presented in Fig. 5, where samples are clustered at each time point and their membership in clusters in tracked over time. Shoe and floor samples showed high stability over time, where most samples cocluster over time in the same clusters (Fig. 5A). Common surfaces had a similar pattern, where common floor samples clustered consistently, while common hand-associated samples could be affiliated with different samples from many individuals (Fig. 5B). To demonstrate how two individuals freely cluster over time, we colored all the samples from two participants (individuals 1 and 29) (Fig. 5C). The samples from individual 1 show that all samples from an individual do not always cluster together, indeed, despite clustering during time points 2 and 4, they cluster separately at time point 2. In contrast, there are two sets of samples from individual 29 that cluster consistently and independently, which reflects the division of samples by type (shoe versus floor) rather than by unique individual.

## DISCUSSION

The use of human microbial signatures as trace evidence remains a young and inexact science. In order for this developing field to become a useful forensic tool, methods will need to be optimized and the myriad factors that influence our microbial interaction with built environments will need to be disentangled. Here, we compared classification methods to link residents to their rooms and personal effects in a common dormitory environment. For classifying individuals, minimum entropy decomposition (MED) was determined to be the best choice based on its high error ratio and ability to recover higher importance scores for all taxa. Further, it appears that the exact sequence variants produced by DADA2 and MED in general are better at identifying individuals than OTU-clustering methods such as UPARSE. This is unsurprising, as exact sequence variants avoid grouping closely related sequences together that could be indicative of individuals. MED is able to recover more diversity within the main skin-associated taxa from the phyla *Proteobacteria*, *Fusobacteria*, *Bacteroidetes*, and *Actinobacteria*, and those sequences are more closely related phylogenetically.

This diversity translates directly into increased utility in classification, as the sequences generated by MED have higher importance scores, and thus discriminative ability, than DADA2 or UPARSE. This is true even at the genus level, indicating that it is able to produce more individual-specific sequences within common skin-associated taxa. At the same time, MED did not recover nearly as many phyla as UPARSE or DADA2 did, and thus underestimated the full diversity of the data set. In this case, fine-scale diversity in highly abundant phyla was what we sought, but it could pose an issue for classification using highly divergent and low-abundance organisms. Further, the increased diversity that MED produces should be interpreted in light of observations that MED can produce false-positive sequence variants in data from mock communities (36, 51).

Here we show that skin-associated samples are useful in linking individuals to rooms that they have inhabited. These microbial signatures appear largely stable, as samples across all time points, spanning a period of 4 weeks, are useful in classification. Temporal stability has been observed in the skin microbiome (15, 17), and this stability in our case extends to the personal samples of each individual. Since individuals contribute their microbial signature to their environment, the presence of a roommate can interfere with classification. There is potential for interaction both between the skin of individuals, which has been seen in couples (20), and a mixing of signatures in the room itself, as seen in examples of cohabitating individuals (3, 19). Roommates were a confounding factor, and classification error linearly correlated with the number of roommates and accounted for 30% of the variance. The increase in classification error should provoke caution in those who seek to discern the signatures of cohabitating individuals, as a mixing of signatures can obscure the true inhabitant of a room.

As in most prior work with microbial forensics (9, 21), our analyses are based on 16S rRNA sequencing methods (52). In contrast, other methods rely on metagenomic markers, which are determined from the sample (22, 24) or known *a priori* (23). Metagenomic methods require shotgun metagenomic sequencing and may be difficult to implement in low-biomass samples or situations where host DNA overwhelms bacterial DNA (53, 54). However, these methods are often significantly more accurate and represent an important direction for future research.

In addition to classification, we were able to examine the larger interaction structure of the college dormitory. The dormitory has two large spheres of interaction, structured by their association with either floors or hands. Floor samples are highly connected to each other and form a dense subgraph in both weighted UniFrac and random forest-based graphs. Close interactions among floor-associated samples may be due to the homogenizing effect of walking, and this has been observed in prior studies (9). In contrast, hand-associated samples appear to be structured both by the individual from which the sample originates and by the common surfaces they interact with. As seen in the weighted UniFrac network analysis, common surfaces form a backbone connecting many separate individuals, which identifies them as potential points of interaction. When looking at the random forest-based analysis, top modules of samples form clusters of interaction that are associated with a number of factors, including whether the samples are hand associated, which floor they originated from, and the sex of the individuals who contributed them. In addition, connections between these top modules are enriched for hands, indicating that hands may be points of interactions between individuals.

When looking at interaction networks, we found that sample clustering over time was highly dependent on sample type, with floor-associated samples showing long, persistent interactions, while common surfaces were more freely interacting. Individual signatures do not always cluster together over time and can form associations over time. While other sources of data, including sexual partners (55) and coauthorship (56), have been used to analyze networks of human interaction (57), this is the first study of which the authors are aware to identify networks of interaction using microbial signatures.

Through individual and common space sampling of a college dormitory, we discovered that MED and other sequence-based methods are superior to those that rely upon OTU clustering. We have also found that common surfaces form a scaffold connecting many individuals, and further, that spheres of interaction are disproportionately connected by hands, indicating them as a means of transmission of microbes. Finally, we have characterized the persistence of interaction and found differences in persistence based on the sample type.

## MATERIALS AND METHODS

### Study design and sample collection.

We collected personal samples from 37 participants in 28 distinct dorm rooms (see Table S1 and S2 in the supplemental material). Samples were collected by swabbing a sterile cotton BD-Swube applicator against the dry surface of interest. Sampling kits were given to study participants for self-sampling with instructions. For the first time point, only the hands of individuals were sampled. The desk, floor, fitted bed sheet, and interior doorknobs of each participant’s room, along with the dominant hand and shoe of the participant, were sampled at three time points after the first time point. The first time point occurred before occupants left for a scheduled school break (end of a quarter) and then immediately upon return. The third and fourth time points were taken 2 and 4 weeks after spring break.

10.1128/mBio.01054-19.8 TABLE S2 Summary of the number of participants on each floor of the dormitory and common surface sampling. Download Table S2, DOCX file, 0.01 MB. Copyright © 2019 Richardson et al. 2019 Richardson et al. This content is distributed under the terms of the Creative Commons Attribution 4.0 International license.

Participants also completed a questionnaire which collected basic information on the subject, the conditions specific to their dorm room, and who they interacted with in their dorm room during the sampling period. This questionnaire was completed each time a set of samples was collected.

Common surfaces were also sampled similarly. Common surfaces specific to the 5th floor included tables in the dormitory lounge, and the handle of the entry door to the lounge. On each floor of the dormitory, the door handles of bathrooms, the floors of each hallway, and the elevator buttons were sampled. Each floor had its own unique combination, and these were swabbed at the same time as personal surfaces.

### Sample processing.

DNA was extracted from each sample using a low biomass variation of the MO BIO Powersoil DNA extraction protocol. 16S rRNA was amplified with the Earth Microbiome Project 16S Illumina Amplicon Protocol (http://www.earthmicrobiome.org/protocols-and-standards/16s/). The V4 region of the 16S rRNA gene was targeted with the 515F-806RB primer pair. Sequencing was performed using an Illumina Miseq sequencer with the protocol described by Caporaso et al. (52).

### Sequence processing.

Each method was processed using the default workflows provided in reference papers given below.

### (i) UPARSE.

Demultiplexed sequences were merged using vsearch v2.3.0 (58) with 10,040,708 successful paired-end reads merged together. Sequences were quality filtered with a maximum expected error of 0.5, with 9,057,613 remaining sequences. Sequences were then dereplicated for 1,276,202 unique sequences. Sequences were then clustered at 97% identity, with 11,658 OTUs and 42,539 chimeras. Sequences were then matched to OTUs with 93.28% of sequences matched to OTUs. A total of 6,011 OTUs passed sequence processing. Chloroplast and mitochondrial DNA was removed, and samples were rarefied to 4,000 counts per sample.

### (ii) MED.

Sequences were processed according to the methods described by Eren et al. (35). Demultiplexed paired-end reads were merged using illumina-utils (59), with Q30 check imposed on sequences, leading to 10,023,266 successfully merged out of 10,023,266 reads. Gaps between sequences were padded with blanks, and samples were decomposed using a -M of 100. A total of 1,732,615 outliers were removed by quality control, and remaining sequences were sorted into 3,748 nodes after refinement. A total of 3,352 passed quality control. Chloroplast and mitochondrial DNA was removed, and samples were rarefied to 4,000 counts per sample.

### (iii) DADA2.

The filtering step of DADA2 version 1.03 was run with no ambiguous base (maxN of 0), maximum expected errors of 2, and quality of truncation of 2. All other commands were run on default settings. Sequences were merged after performing quality filtering. After merging, 34,043 sequences were observed, and 18,329 sequences were not chimeras. A total of 4,307 unique sequences passed final quality filtering. Chloroplast and mitochondrial DNA was removed, and samples were rarefied to 4,000 counts per sample.

### Taxonomic identification.

All sequences were taxonomically identified using the same implementation of RDP (60) implemented in DADA2 to enable comparison between the sequencing methods. Taxonomy was assigned using the SILVA (61) training set version 123.

### Phylogenetic trees.

Sequences were aligned with the R package *MSA* (62) version 1.4.5, using the Muscle (63, 64) algorithm. Phylogenetic trees were then generated using the R package *Phangorn* (65) version 2.1.1. The tree was first created by neighbor joining and fitted with GTR clock model.

### Data analysis and visualization.

Data cleaning and shaping were performed using R 3.3.2-R3.5.2 and the packages *dplyr* 0.7.8 (66) and *reshape2* 1.4.3 (67). Visualization and analysis were performed using *phyloseq* 1.26 (68), igraph v1.2.4.1 (69), ggnetwork (70), and *ggplot2* v3.1.1 (71). Boxplots with significance were generated using *ggpubr* v0.2 (72). Phylogenetic distance was calculated using the “cophenetic.phylo” function from *ape* v5.3 (73). Differential abundance calculations were performed using DESeq2 v1.12.4 (74). Diversity measures were calculated using *vegan* v2.5-4 (75). Ideas for analysis, along with basic code snippets were taken from Callahan et al. (76). Community clustering was performed using the Infomap (77, 78) and alluvial diagrams were generated using the “Map & Alluvial Generator” (http://www.mapequation.org/apps/MapGenerator.html).

### Random forests.

Random forest models were generated using *randomForest 4.6-13* (79) and *ranger* v0.10 (80). For classification of individuals, all room samples for individuals were used to predict the hands of individuals. This was repeated 10 times for cross validation (CV-10), and proximities/importance scores were averaged across runs. For comparisons of men versus women, all personal samples were subdivided into testing/training sets, with two thirds of samples in the training set and one third in the testing set. This was run thrice (CV-3).

### Ethics.

This study (institutional review board [IRB] number IRB15-0373) was approved by Biological Sciences Division (BSD) IRB Committee A of The University of Chicago Biological Sciences Division/University of Chicago Medical Center.

### Data availability.

Sequencing data and sample data are available from Qiita, study ID 12470, and from EBI, project number PRJEB33050/ERP115809. Phyloseq object files from each of the three sequencing methods, along with sequence tables, taxonomy tables, anonymized sample data, and phylogenetic trees are available on Github at https://github.com/MiPZR/Dorm-Microbiome.
