# Supplementary material for: Microbial Similarity between Students in a Common Dormitory Environment Reveals the Forensic Potential of Individual Microbial Signatures
Source: mBio. 2019 Jul 30;10(4):e01054-19. doi: 10.1128/mBio.01054-19 (PMC6667619; doi:10.1128/mBio.01054-19)
Supplement: TABLE S1 [file mBio.01054-19-st001.docx]

| Method | UPARSE | DADA2 | MED |
| --- | --- | --- | --- |
| OTUs/Sequences | 6011 | 4307 | 3352 |
| Phyla | 25 | 23 | 9 |
| Class | 56 | 49 | 24 |
| Order | 121 | 103 | 62 |
| Family | 346 | 267 | 153 |
| Genus | 1002 | 747 | 440 |
| Species (only assigned for exact 16S match) | 605 | 937 | 881 |
| Average Phylogenetic Distance | 2.62 | 2.27 | 1.36 |
