# Supplementary figures and images for: Microbial Similarity between Students in a Common Dormitory Environment Reveals the Forensic Potential of Individual Microbial Signatures
Source: mBio. 2019 Jul 30;10(4):e01054-19. doi: 10.1128/mBio.01054-19 (PMC6667619; doi:10.1128/mBio.01054-19)

Accuracy of Room Surfaces Predicting Hands

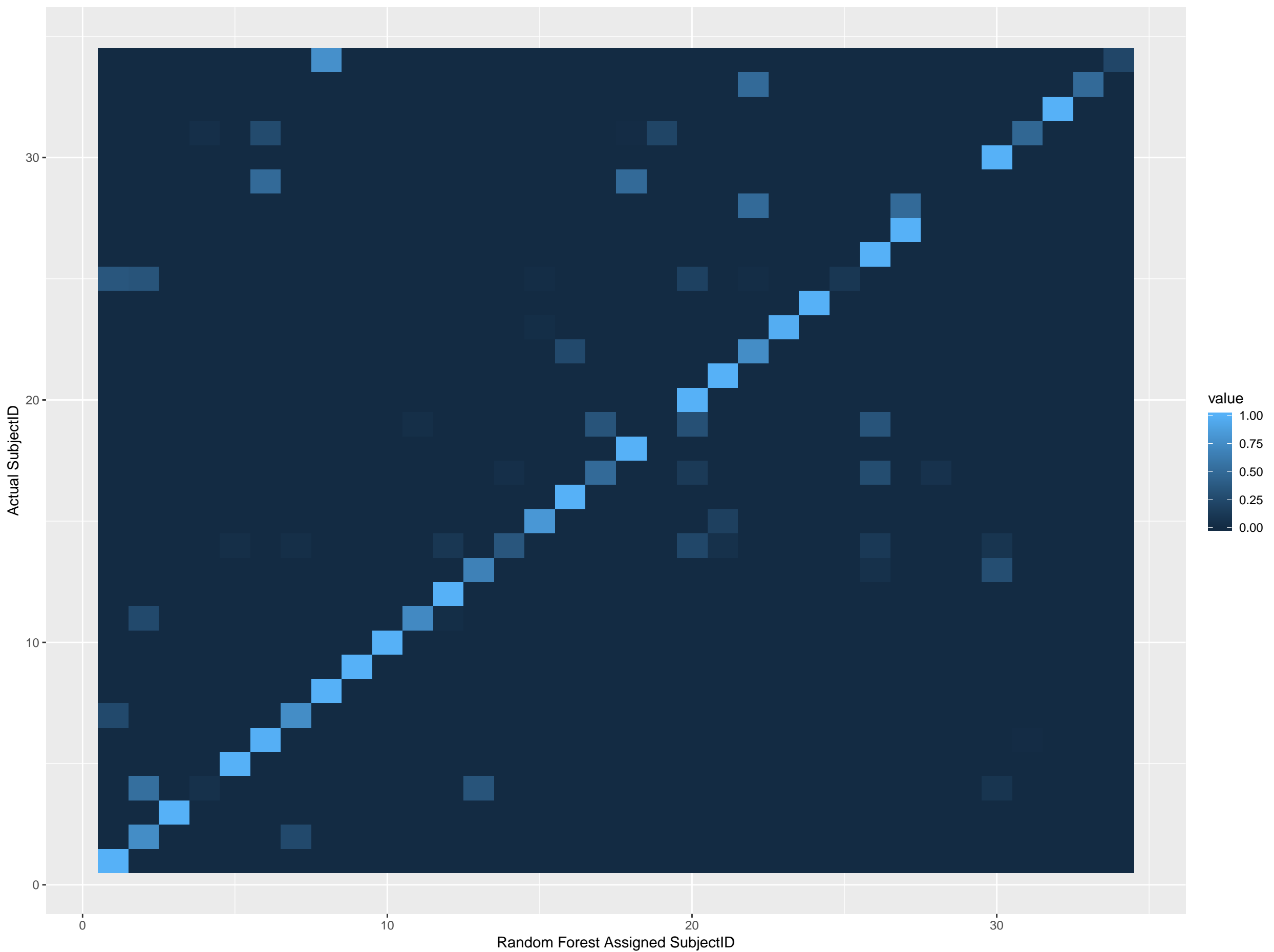

Supplement: FIG S1 [file mBio.01054-19-sf001.pdf]

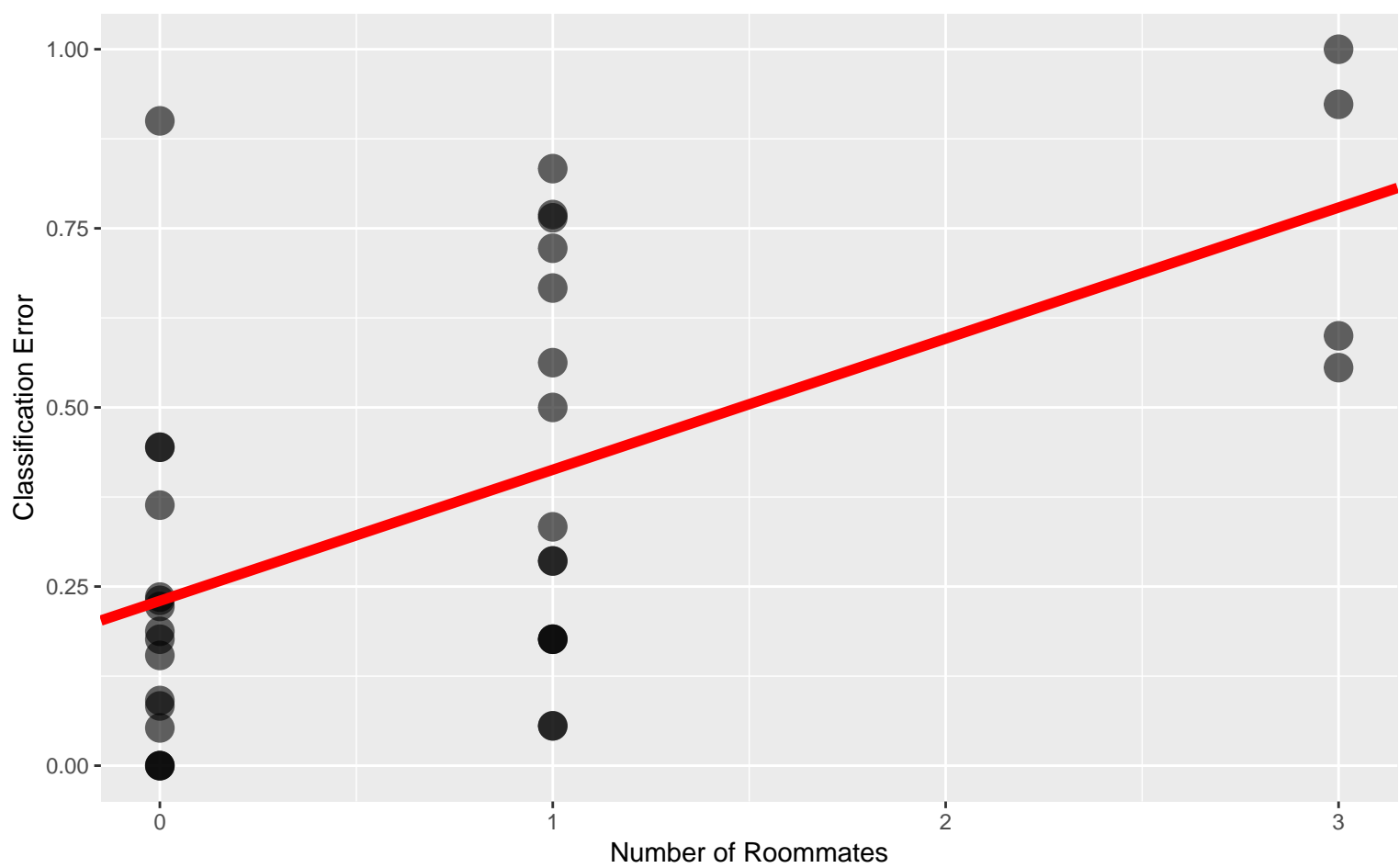

Supplement: FIG S2 [file mBio.01054-19-sf002.pdf]

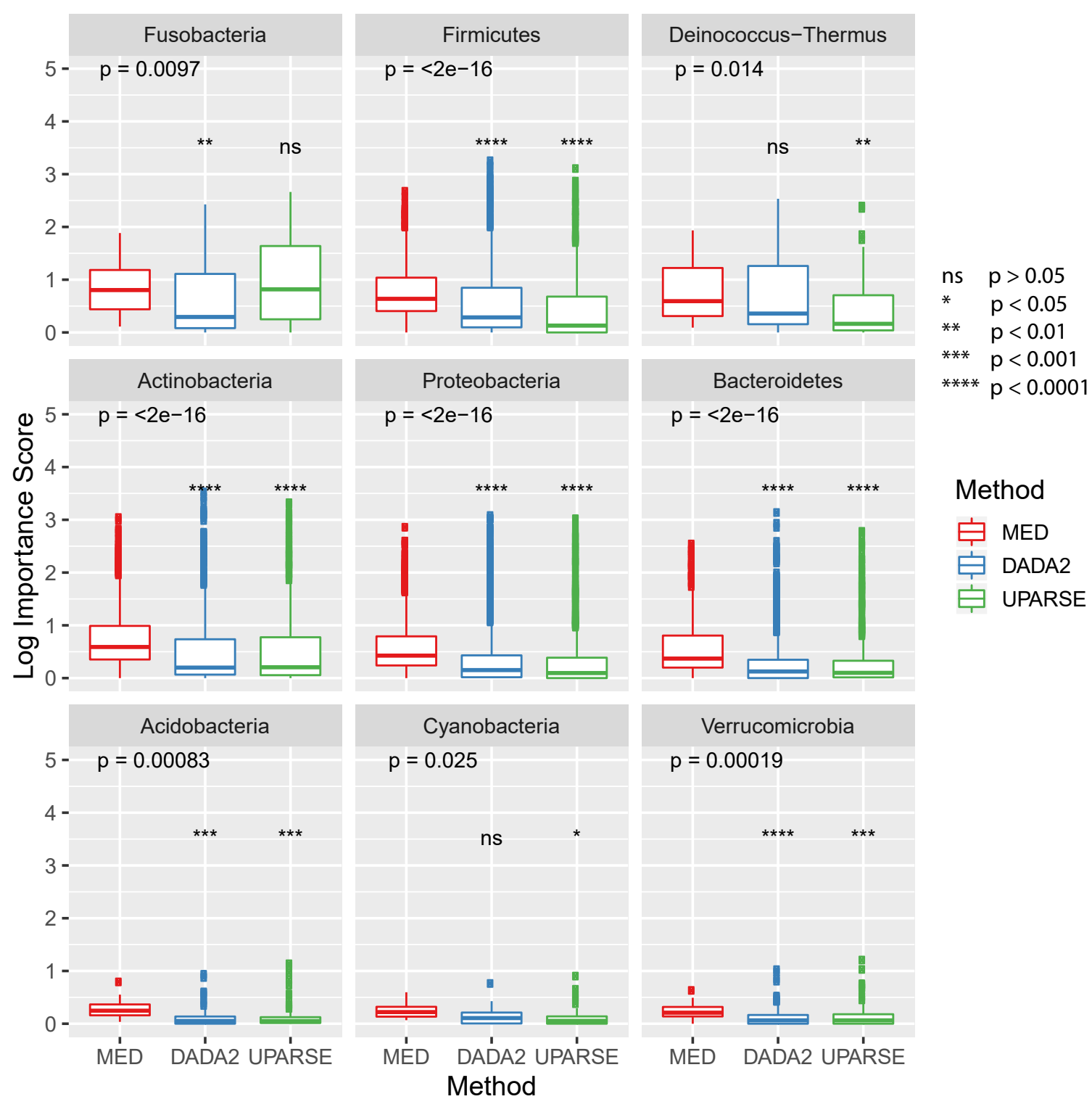

Supplement: FIG S3 [file mBio.01054-19-sf003.pdf]

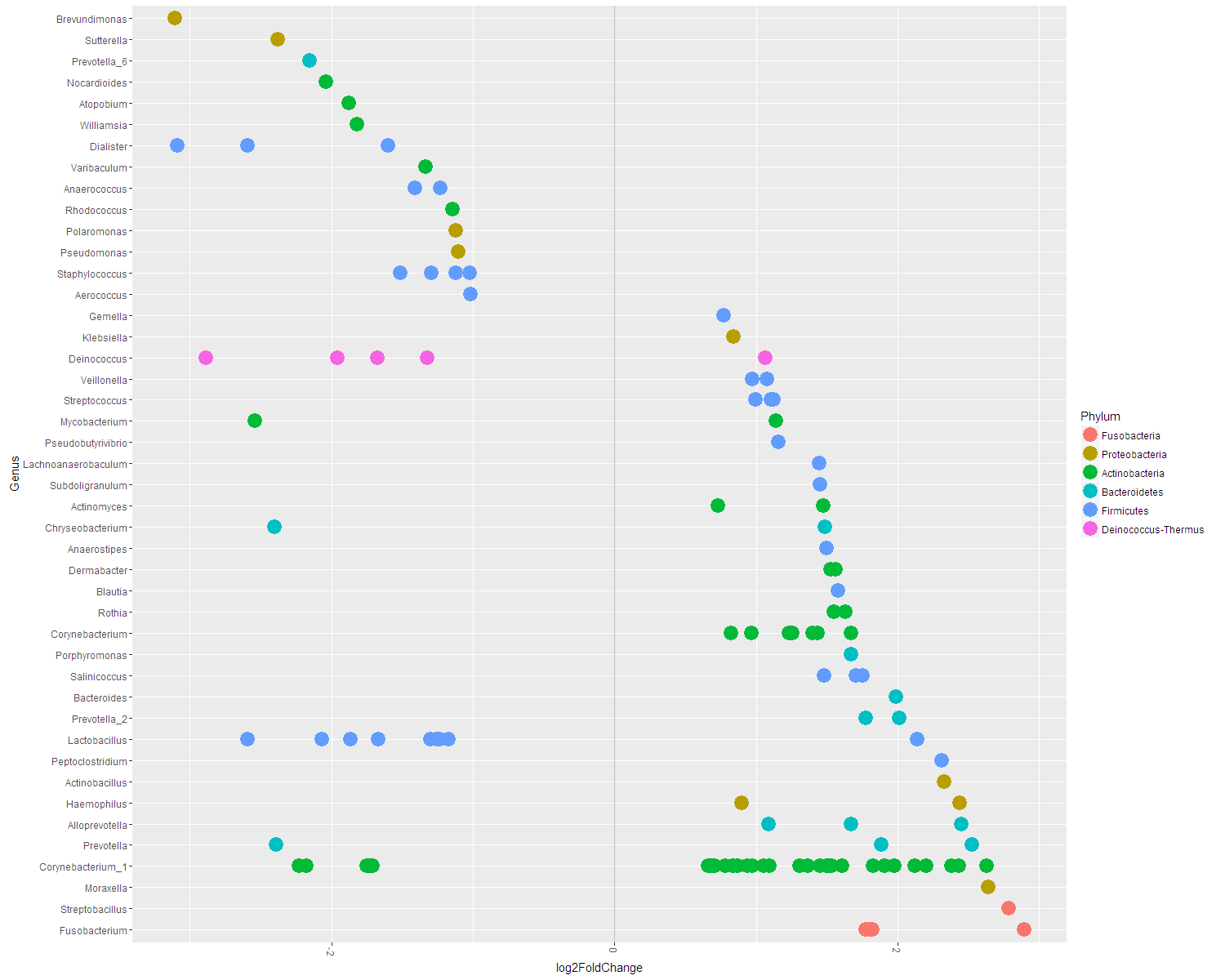

Supplement: FIG S4 [file mBio.01054-19-sf004.tif]

**A**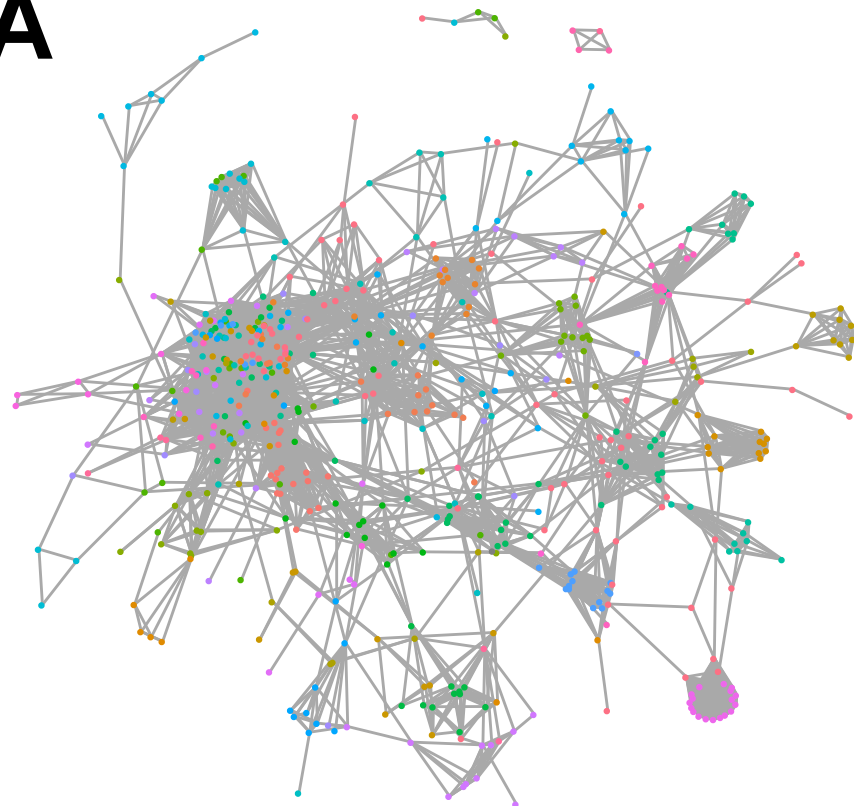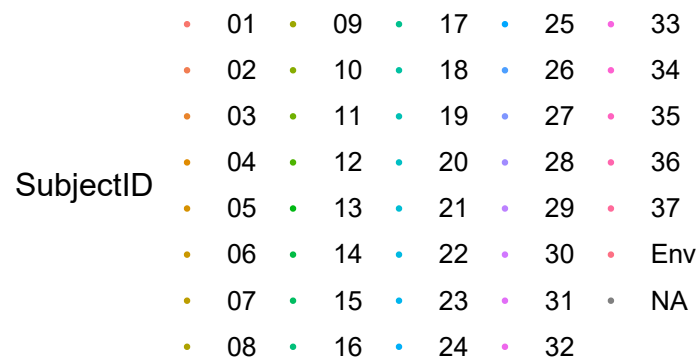**B**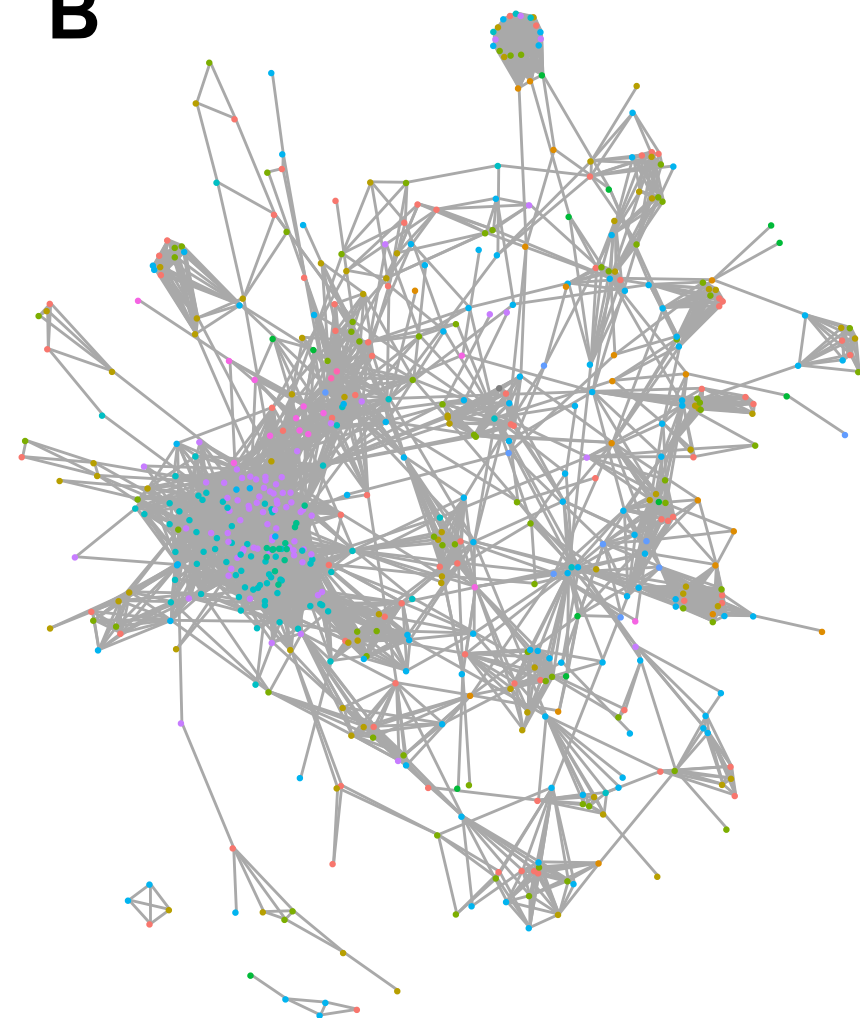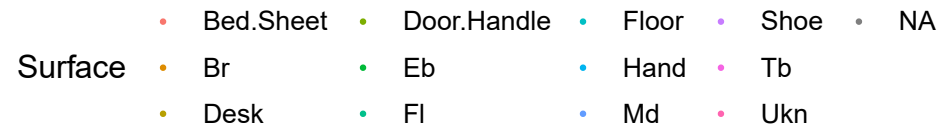

Supplement: FIG S5 [file mBio.01054-19-sf005.pdf]

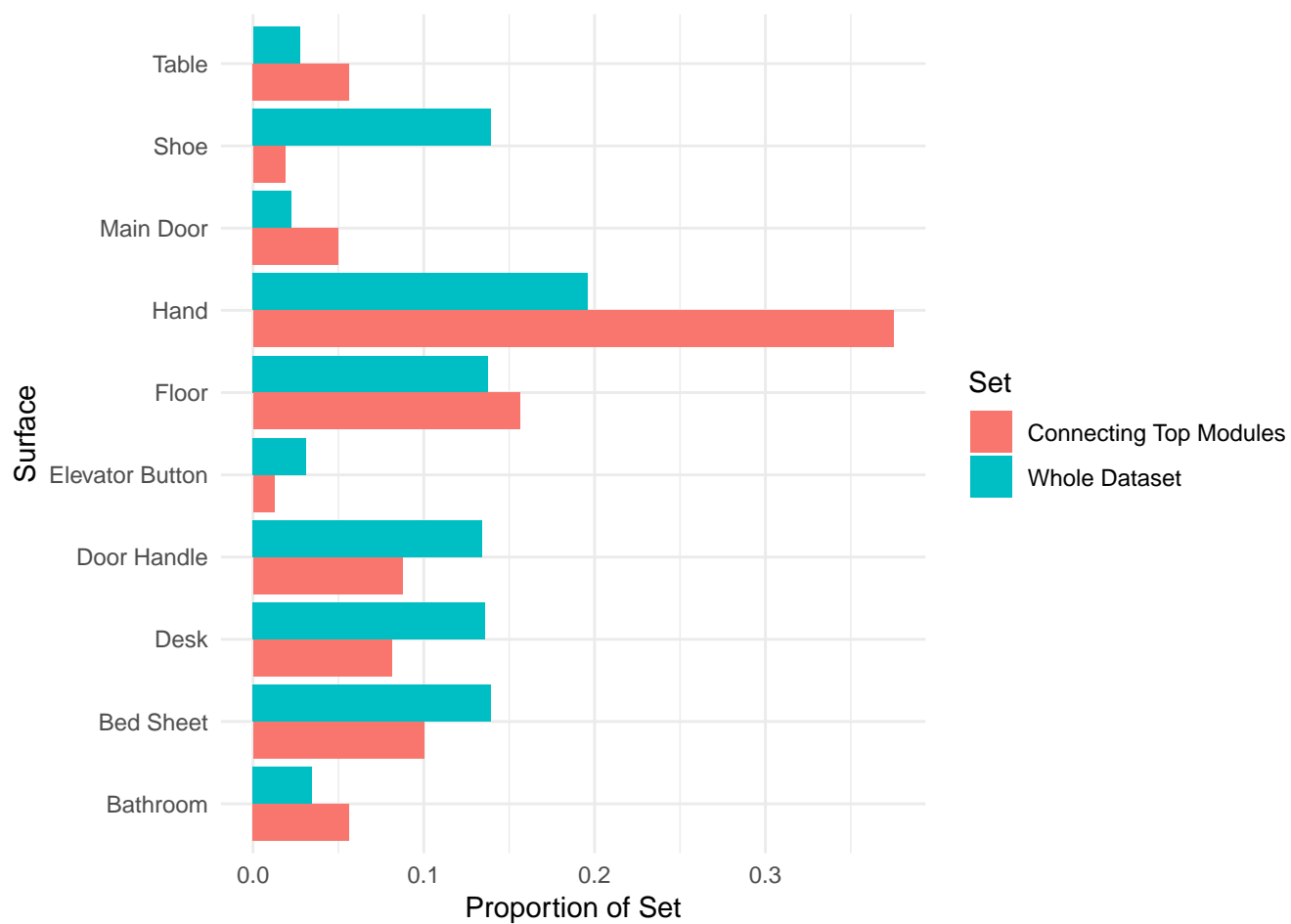

Supplement: FIG S6 [file mBio.01054-19-sf006.pdf]
