# Supplementary material for: Microbial Similarity between Students in a Common Dormitory Environment Reveals the Forensic Potential of Individual Microbial Signatures
Source: mBio. 2019 Jul 30;10(4):e01054-19. doi: 10.1128/mBio.01054-19 (PMC6667619; doi:10.1128/mBio.01054-19)
Supplement: TABLE S2 [file mBio.01054-19-st002.docx]

| Floor | Number of Participants | Bathroom Door Handle | Hallway Floor | Elevator Buttons | Miscellaneous |
| --- | --- | --- | --- | --- | --- |
| 1 | 0 | No | No | No | Entry Door |
| 5 | 11 | Male and Unisex | Yes | Yes | 3 Common Tables |
| 6 | 5 | Female | Yes | Yes |  |
| 7 | 9 | Male | Yes | Yes |  |
| 8 | 12 | Female | Yes | Yes |  |
